# Understanding inter-individual variability in pharmacokinetics/pharmacodynamics of aripiprazole in children with tic disorders: Individualized administration based on physiological development and CYP2D6 genotypes

**DOI:** 10.3389/fphar.2022.1048498

**Published:** 2022-12-02

**Authors:** Yingying Xin, Liuliu Gao, Yali Tuo, Gang Nie, Yan Mei, Chen Chen, Jun Wang, Sichan Li, Dan Sun, Qiaoqiao Qian, Yongli Fu, Yang Wang, Zhisheng Liu

**Affiliations:** ^1^ Department of Neurology, Wuhan Children’s Hospital, Tongji Medical College, Huazhong University of Science and Technology, Wuhan, China; ^2^ Department of Pharmacy, Wuhan Children’s Hospital, Tongji Medical College, Huazhong University of Science and Technology, Wuhan, China; ^3^ Department of Pharmacy, Union Hospital, Tongji Medical College, Huazhong University of Science and Technology, Wuhan, China; ^4^ Department of Pharmacy, Wuhan Mental Health Center, Tongji Medical College, Huazhong University of Science and Technology, Wuhan, China

**Keywords:** pediatric, tic disorders, aripiprazole, CYP2D6, population pharmacokinetics, pharmacodynamics

## Abstract

**Objective:** This study aims to develop a combined population pharmacokinetic (PPK) model for aripiprazole (ARI) and its main active metabolite dehydroaripiprazole (DARI) in pediatric patients with tic disorders (TD), to investigate the inter-individual variability caused by physiological and genetic factors in pharmacokinetics of ARI and optimize the dosing regimens for pediatric patients.

**Methods:** A prospective PPK research was performed in Chinese children with TD. Totally 84 patients aged 4.83–17.33 years were obtained for the pharmacokinetic analysis. 27 CYP2D6 and ABCB1 gene alleles were detected. Moreover, the clinical efficacy was evaluated according to reduction rate of Yale Global Tic Severity Scale (YGTSS) score at the 12th week comparing with the baseline. Monte Carlo simulations were used to evaluate and optimize dosing regimens.

**Results:** The PPK model was established to predict the concentrations of ARI and DARI. Body weight and CYP2D6 genotype were the significant covariates affecting the clearance of ARI. The DARI/ARI metabolic ratios (MRs) of AUC_24h_, C_min_ and C_max_ at the steady state of results were ultra-rapid metabolizers (UMs) > normal metabolizers (NMs) > intermediated metabolizers (IMs). MRs could be used to distinguish UMs or IMs from other patients. The best predictor of clinical efficacy for TD was the trough concentration of ARI and the cut-off point was 101.636 ng/ml.

**Conclusion:** The pharmacokinetics of ARI and DARI in pediatric TD were significantly influenced by body weight and CYP2D6 genotype. Individualized dosing regimens were recommended for pediatric patients with TD to ensure clinical efficacy.

## Highlights

1) A population pharmacokinetic model was firstly established in pediatric tic disorders.
2) Body weight and CYP2D6 genotype were the significant covariates.
3) Dehydroaripiprazole/aripiprazole ratios could be substituted for CYP2D6 genotyping.
4) Trough concentration of aripiprazole could predict clinical efficacy of tic disorders.
5) Precise dosing regimens were proposed based on body weight and CYP2D6 genotype.

## Introduction

Tic disorders (TD) is one of the most common neurodevelopmental disorders in childhood, which is characterized with sudden, rapid, recurrent, nonrhythmic motor movement or vocalization (Deeb et al., 2019). The Diagnostic and Statistical Manual of Mental Disorders, 5th edition (DSM-5) classifies TD into provisional tic disorders (PTD), chronic motor or vocal tic disorders (CTD) and Tourette syndrome (TS) (Association AP, 2013). According to the national-scale psychiatric epidemiological survey in China in 2021, the prevalence of PTD, CTD, TS in school children and adolescents is 1.2%, 0.9% and 0.4%, respectively (Li et al., 2021). Before treatment, Yale Global Tic Severity Scale (YGTSS) is commonly used to assess the frequency and severity of tic symptoms in clinics (Martino et al., 2017). The treatment of TD contains pharmacological treatment and non-pharmacological treatment and patients with moderate to severe conditions need pharmacological treatment. Tiapride, clonidine adhesive patch and aripiprazole (ARI) are recommended as the first-line pharmacological treatment in the expert consensus (Liu et al., 2020). Recently, the European Society for the Study of Tourette Syndrome (ESSTS) proposed that ARI should be the first choice of drug for TD both in children and adults (Roessner et al., 2022).

ARI is the third-generation atypical antipsychotic which acts as a partial agonist at the dopamine D2, dopamine D3 and serotonin 5-HT1A receptors and an antagonist at the serotonin 5-HT2A receptors (Shapiro et al., 2003). The main active metabolite of ARI is dehydroaripiprazole (DARI), which accounts for 40% ARI exposure in plasma (Kinghorn and Mcevoy, 2005). Due to the long half-life (T_1/2_) of ARI, it usually reaches steady state concentrations after 14 days of treatment or dose adjustment (Prommer, 2017). According to the 2017 Arbeitsgemeinschaft für Neuropsychopharmakologie und Pharmakopsychiatrie (AGNP)-therapeutic drug monitoring (TDM) expert group consensus guidelines for TDM recommendations of ARI, the therapeutic reference ranges are 100–350 ng/ml for ARI, and 150–500 ng/ml for ARI plus DARI (Hiemke et al., 2018). For pediatric patients, the application of TDM in ARI appears to be more important in terms of their development (Rafaniello et al., 2020). However, there are no specific therapeutic reference ranges in ARI or DARI for children with TD. Meanwhile, a few related literatures were reported just in adult patients with schizophrenia (Kirschbaum et al., 2008; Lin et al., 2011).

ARI is mainly metabolized in the liver through three biotransformation pathways: dehydrogenation, hydroxylation and N-dealkylation *via* CYP2D6 and CYP3A4 enzyme (Belmonte et al., 2018). ARI is metabolized to a lesser extent by CYP3A4 enzyme, recent research progress showed that CYP3A4 genotype did not significantly influence the pharmacokinetics of ARI and DARI (Belmonte et al., 2018; Saiz-Rodríguez et al., 2020). The ATP-binding cassette sub-family B member 1 (ABCB1) gene is located on chromosome 7 at q21 and codes for the P-glycoprotein (P-gp) (Gottesman et al., 1995). P-gp involves in the process of absorption, distribution and elimination of ARI, which transports ARI across intracellular and extracellular membranes (Thiebaut et al., 1987). Both ARI and DARI are possible substrates of P-gp and it was reported that ABCB1 polymorphism was associated with the serum concentrations of ARI or DARI (Gunes et al., 2008; Rafaniello et al., 2018). CYP2D6 is a highly polymorphic gene. According to the classifications of CYP2D6 genotype, subjects are usually divided into four phenotypes as following: poor metabolizers (PMs), intermediated metabolizers (IMs), normal metabolizers (NMs), and ultra-rapid metabolizers (UMs) (Gaedigk et al., 2017; pharmvar, 2022). Previous studies showed that there was high variability in inter- and intra-individual pharmacokinetics of ARI (Molden et al., 2006), which could be mainly explained by CYP2D6 genetic polymorphisms (Jovanovic et al., 2020). Those studies were mainly aimed at psychiatric patients or healthy subjects in adults. However, for TD patients, especially in children, there were insufficient research data and several limitations. Besides the genetic polymorphisms, the other factors such as physiological developmental index, liver and renal function, or drug combination may influence the pharmacokinetics of ARI. Moreover, the correlation between serum concentrations and treatment response of ARI is still unclear and the therapeutic range of serum concentrations has not been estimated. Furthermore, the dose of ARI is often adjusted on the basis of drug response and side effects in clinics and the optimal dose regimens are under researched.

In this study, a combined population pharmacokinetic (PPK) model of ARI and its metabolite DARI was developed. It investigated the contributions of CYP2D6 and ABCB1 genetic polymorphisms, physiological factors, and drug combinations in ARI and DARI pharmacokinetics. Finally, the precise medication for pediatric patients with TD was promoted and appropriate dosing regimens were proposed.

## Materials and methods

### Study population

This prospective study was conducted at the Department of Pediatric Neurology of Wuhan Children’s hospital, Huazhong University of Science and Technology, from January 2021 to July 2022. The inclusion criteria for patients included in the pharmacokinetic study were listed as follows: 1. The diagnosis complies with the diagnostic criteria of TD in DSM-5; 2. Chinese patients, aged less than 18 years, no matter of sex 3. Pharmacological treatment included ARI monotherapy or ARI add-on therapy; 4. Free from any known organic diseases, normal liver and renal functions, normal electrocardiogram (ECG) and so on. The exclusion criteria for pharmacokinetic study were listed as follows: 1. Subjects participated in other clinical trials in the past 1 month; 2. the clinical data were incomplete; 3. Poor medication adherence or lost follow-up; 4. Patients with epilepsy, encephalitis, schizophrenia, abnormal liver function or other organic diseases. The inclusion criteria for pharmacodynamic study were listed as follows on the basis of above inclusion criteria in pharmacokinetic study: YGTSS total score≥25 at the baseline. The exclusion criteria for pharmacodynamic study were as follows on basis of the above exclusion criteria in pharmacokinetic study: add other drugs for the treatment of TD or adjust the dose of drugs other than ARI during follow-up. The study was approved by the Ethics Committee of Wuhan Children’s hospital, Huazhong University of Science and Technology (No: 2021R101-E01). Informed consent was obtained from the guardians of the children involved in this study.

### Dosage regimen, blood sampling and data collection

ARI was orally administered to the pediatric patients at a dose of 1.25–5 mg once a day in the first week, with a gradual increase to the target dose of 2.5–20 mg/d. The dose adjustment interval was at least 1 week. A sampling strategy was selected to collect blood samples for pharmacokinetic analysis. The dosing and sampling time was accurately recorded. Blood samples (4 ml) were collected from patients after orally taking ARI for at least 14 consecutive days. The serum concentrations of ARI were tested, subsequently the residual blood samples were separated and stored at -70°C for CYP2D6 and ABCB1 genetic testing.

Individual laboratory and demographic parameters were collected from the electronic medical records database, including age, gender, height, weight, alanine aminotransferase (ALT), aspartate aminotransferase (AST), direct bilirubin (DBIL), total bilirubin (TBIL), albumin (ALB), globulin (GLB), γ-glutamyltranspeptidase (γ-GT), blood urea nitrogen (BUN), serum cystatin C (Cys-C), and serum creatinine concentration (Scr). The body surface area (BSA) was calculated by the Mosteller formula. The estimated glomerular filtration rate (eGFR) was obtained by the modified Schwartz formula.

### Quantification of ARI and DARI

The concentrations of ARI and DARI were detected by using the HPLC method. The preparation procedures of the sample were as follows: Blood sample was centrifuged at 1,500 g for 10 min and 0.5 ml serum sample was added into the solid-phase extraction column (Agela Technologies, Cleanert ODS C18), subsequently methanol containing 0.05% hydrochloric acid was used for elution. The Innoval C18 column (Agela Technologies, 5 μm, 4.6 × 250 mm) was used for separation. Ammonium acetate (0.03 mol/L): methanol = 20:80 was prepared as the mobile phase. The wavelength of ultraviolet (UV) detection was 217 nm. The linear ranges of ARI and DARI detection were 10–1,490 ng/ml and 15–1,070 ng/ml, respectively. The intra- and inter-day precisions for ARI and DARI were within 10%.

### CYP2D6 and ABCB1 SNP detection, CYP2D6 allele and genotype frequencies

First-generation sequencing was utilized for genetic detection, consisting of DNA extraction, polymerase chain reaction (PCR) amplification and SNP detection process. DNA extraction was performed by MolPure^®^ blood DNA kits (Yeasen Biotechnology, Shanghai). SNP detection was determined by 3730XL Sequencer (ABI, Inc., USA) by Sangon Biotech (Shanghai, China).

A total of 27 SNPs were detected and consisted of 25 SNPs of CYP2D6 and 2 SNPs of ABCB1, including CYP2D6*3 (rs35742686), CYP2D6*4 (rs3892097), CYP2D6*6 (rs5030655), CYP2D6*9 (rs5030656), CYP2D6*14 (rs5030865), CYP2D6*17 (rs28371706), CYP2D6*33 (rs28371717), CYP2D6*35 (rs769258), CYP2D6*41 (rs28371725), CYP2D6*49 (rs1135822), CYP2D6*51 (rs72549348), CYP2D6*54 (rs267608297), CYP2D6*69 (rs267608289), rs1135840, rs16947, rs1058164, rs28371705, rs28371703, rs28371702, rs28371699, rs29001518, rs1080995, rs1065852, rs1080989, rs1080985, ABCB1 C3435T (rs1045642) and G2677T/A (rs2032582). The frequencies of CYP2D6 allele mutation were shown in gene heat map.

Based on the classification standard (Zhang et al., 2021), patients were categorized into four CYP2D6 metabolizer phenotypes. UMs were defined as carrying more than 2 normal function alleles, eg *1*1, *1*2, *2*2. NMs were defined as carrying 1 functional allele, e.g., *1*10, *1*33. IMs were defined as carrying 1 decreased-function allele and 1 non-function allele or carrying 2 decreased-function alleles, e.g., *3*10, *10*10. PMs were defined as carrying 2 non-function alleles, e.g., *3*3.

### Clinical efficacy observation

Outpatients and telephone follow-ups were conducted respectively at the baseline and after 12 weeks of ARI treatment. The basic information such as gender, age, height, weight, disease type, comorbidity, dosage, concomitant medication, liver and kidney function, serum concentrations of ARI and metabolite DARI, clinical efficacy was recorded in detail during follow-up. The severity of TD was assessed by Yale Global Tic Severity Scale (YGTSS). The scale includes three parts: motor tic score, vocal tic score and functional impairment score. The sum of the three parts is the total scores of YGTSS. The higher the total scores, the more serious the symptoms of disease. After 12 weeks treatment, the clinical efficacy was assessed by the YGTSS score reduction rate (%). YGTSS score reduction rate (%) = (total scores before treatment–total scores after treatment)/total score before treatment × 100%. Compared with the baseline YGTSS scores, when YGTSS score reduction rate is less than 50%, it is evaluated as ineffective. When YGTSS score reduction rate is more than 50%, it is evaluated as effective. Side effects or adverse events were observed and recorded during treatment.

### PPK modeling

The population pharmacokinetics analysis was performed by the software Phoenix^®^ NLME (Version 8.2.0. 4383, Pharsight Corporation, USA) and R program (Version 4.0.2). The first order conditional estimation-extended least squares (FOCE ELS) method was applied to the estimation of population pharmacokinetic parameters and the variabilities.

### Construction of the base model

One- or two-compartment model with first-order elimination were evaluated for ARI and DARI. Since the lack of absorption and distribution phase data, the parameters were difficult to be estimated by two-compartment model. Therefore, two one-compartment tandem models were applied in the construction of the base model for both ARI and DARI. According to the reports in the literature, the absorption rate constant (*k*_*a*_) was fixed at 1.06 h^−1^ (Jovanovic et al., 2020), and the bioavailability (*F*) was not estimated. The material balance formulas were shown in Equations 1-3.

$$dA\left(dose\right)/dt=-k_{a}\times A\left(dose\right)\times F$$ (1)

$$dA\left(p\right)/dt=k_{a}\times A\left(dose\right)\times F-CL/Vd\times A\left(p\right)$$ (2)

$$dA\left(m\right)/dt=F_{m}\times k_{n}\times CL/V_{d}\times A\left(p\right)-{CL}_{\left(m\right)}/{Vd}_{\left(m\right)}\times A_{\left(m\right)}$$ (3)

where *A(dose)* represents the dosage of ARI, *k*_*a*_ is the absorption rate constant, *F* is the oral bioavailability of ARI, *A(p)* is the amount of ARI in the central compartment, *CL/F* is the apparent clearance of ARI, *Vd/F* is the apparent volume of distribution, *A(m)* is the amount of DARI in the metabolic compartment, *Fm* is the dosage conversion fraction from ARI to DARI, since the amount of ARI translate to DARI was unknown, *Fm* was not estimated in this study. *kn* is the molecular mass ratio of DARI/ARI (0.995), *CL(m)/Fm* is the apparent clearance of DARI, *Vd(m)/Fm* is the apparent volume of distribution of DARI.

The exponential and proportional model were respectively applied to the estimation of inter- and intra-individual variability, which were shown as Equations 4, 5.

$$Pi=\theta \times \exp\left(\eta i\right)$$ (4)

$$Y=IPRED\times \left(1+\varepsilon \right)$$ (5)

where *Pi* is regarded as the individual pharmacokinetic parameter, *θ* represents the typical value of the population pharmacokinetic parameter, *ηi* is the inter-individual variation, and *ηi* conforms to a normal distribution with mean 0 and variance ω^2^. *Y* is the observed drug concentration, *IPRED* is the individual prediction, *ε* is the intra-individual variation, and *ε* conforms to a normal distribution with mean 0 and variance σ^2^.

### Covariate analysis

After the construction of the basic structure model, the stepwise method was used to investigate the influence of covariates on the pharmacokinetic parameters, and the inclusion or exclusion of a covariate depended on the changes of the objective function value (OFV). The forward and backward selections were utilized. In the forward selection, the covariate would be added into the basic structure model if the decrease in OFV was more than 3.84(*p* < 0.05, df = 1). The covariates were removed from the model one by one. In the backward selection, the covariate should be removed if the increase of OFV was less than 6.64 (*p* < 0.01, df = 1). Then the final PPK model was established.

The covariates in the final model consisted of continuous variables and categorical variables. Continuous variables included age, height, weight, liver and kidney function indicators. Categorical variables included gender, genotype, and combination medication. The introduction methods of continuous and categorical variables were shown in Equations 6, 7, respectively. Five developmental models were tried to analyze the effect of physiological development on ARI clearance (Ding et al., 2015; Li et al., 2020).

$$\theta_{i}=\theta \times {\left(\frac{Cov\_j}{Cov\_median}\right)}^{\theta_{cov}}$$ (6)

$$\theta_{i}=\theta \times \exp\left(\theta_{cov}\right)$$ (7)

where *θ*_*i*_ represents the population predicted value of pharmacokinetic parameter, *θ* is the population typical value, *Cov*_*-*_ *j* is the *j*th continuous covariate, *Cov-median* is the median value of the covariate, and *θcov* is regarded as the fixed effect of the covariate on the parameter.

### Validation of the final model

The prediction performance of the population pharmacokinetic model was usually evaluated by nonparametric bootstrap analysis, goodness-of-fit plots, normalized prediction distribution errors (NPDE), and visual predictive check (VPC). Both bootstrap analysis and VPC tests were run for 1,000 times to assess the accuracy and stability of the final model established in this study. To confirm the reliability of the final model, the bootstrap parameters were required to be within 10% of the final model parameters and the 90% CIs of the VPC predicted values should cover most of the measured values. Goodness-of-fit plots were employed to check the agreement between the predicted and observed value, to verify whether the prediction error has a significant drift with the predicted value or observation time, including observed concentrations vs. individual predictions or population predictions (PRED), conditional weighted residuals (CWRES) vs. PRED or time. The distributional trends and characteristics of the data error were checked by NPDE. The results were generalized graphically by default as obtained from the R package, including Quantile-quantile plot, the NPDE histogram, and scatterplots of NPDE against time after the last dose or against PRED. The NPDE was expected to follow the normal distribution.

### Model-based simulations

The area under the curves over 24 h (AUC_24h_), peak concentrations (C_max_) and trough concentration (C_min_) of ARI and DARI at steady-state were calculated by employing Bayesian maximum posterior probability method. The metabolic ratio (MR, DARI/ARI) was calculated and the comparison of MRs between different groups (UMs, NMs, IMs) was explored. The ROC curve was used to find the diagnostic cut-off point of MR for distinguishing IMs or UMs from other patients. The relationship between steady-state trough concentration of ARI, DARI, ARI plus DARI and clinical efficacy was investigated respectively by ROC method. Otherwise, the relationship between the dose of ARI and clinical efficacy was investigated and the diagnostic cut-off point was determined. The concentrations collected at non-standard valley time point were corrected by Bayesian method.

The target indexes of the simulated trough concentration of ARI and ARI plus DARI were determined on the basis of the observation of clinical efficacy and the standard of AGNP. The Monte Carlo simulations were performed by the parameters and variabilities derived from the final model. Each dose, as well as each scenario, was simulated for 1,000 times to evaluate the clinical efficacy and safety of different dosing regimens under different covariate factors. The steady-state trough concentration and the probability of reaching the target (PTA) of efficacy and toxicity were calculated.

## Results

### Study population

Totally 84 patients aged 4.83–17.33 years old were included for PPK analysis. These patients consisted of 64 males and 20 females, all of which were diagnosed with TD. The body weight ranged from 17.90 to 100 kg. The demographic and clinical characteristics of the patients for the pharmacokinetics analysis in this study are summarized in Table 1.

**TABLE 1:** Basic information of patients for the pharmacokinetics analysis in this study.

	n	Mean ± SD	Median (range)
Patients	84	—	—
Sex (male: female)	64:20	—	—
Age (year)	—	9.62 ± 2.73	9.17 (4.83–17.33)
Body weight (kg)	—	40.25 ± 16.76	36.00 (17.90–100.00)
Height (cm)	—	141.92 ± 15.85	142.00 (108.00–176.00)
Body surface area (m^2^)	—	1.24 ± 0.32	1.21 (0.73–2.21)
ALT (U/L)	—	14.67 ± 7.81	13.00 (5.00–50.00)
AST (U/L)	—	21.47 ± 4.60	21.00 (12.00–33.00)
DBIL (mol/L)	—	3.84 ± 1.36	3.60 (1.90–8.40)
TBIL (μmol/L)	—	9.39 ± 3.92	8.65 (3.00–23.70)
γ-GT (U/L)	—	13.14 ± 4.99	12.00 (7.00–40.00)
GLB (g/L)	—	23.22 ± 3.15	23.35 (15.40–30.00)
ALB (g/L)	—	48.51 ± 2.39	48.25 (43.10–55.30)
CRE (μmol/L)	—	43.77 ± 10.29	42.05 (28.00–91.70)
BUN (mmol/L)	—	4.54 ± 0.99	4.59 (2.20–7.60)
Cys-C (mg/L)	—	0.86 ± 0.11	0.86 (0.62–1.25)
eGFR (mL/min·1.73m^2^)	—	121.72 ± 18.58	121.56 (66.49–162.39)
Concentration of ARI (ng/ml)	—	137.11 ± 81.47	123.96 (11.31–346.55)
Concentration of DARI (ng/ml)	—	50.83 ± 29.39	44.10 (15.19–162.40)
Number of combined medication cases			
Tiapride	26	—	—
Clonidine	41	—	—
Methylphenidate	1	—	—
Topiramate	3	—	—
Trihexyphenidate	1	—	—
Inosine	4	—	—
Chinese traditional medicine	10	—	—

### Phenotypic and CYP2D6 allele frequencies

All subjects were genotyped for all the variants (n = 84 subjects). The results of CYP2D6 genotype and phenotype were shown in Table 2. The results of CYP2D6 allele mutation frequencies were shown in Supplementary Figure S1 in the supplementary document.

**TABLE 2:** The distribution of CYP2D6 genotypes and phenotypes.

Genotype/Phenotype	N (%)	Genotype/Phenotype	N (%)
CYP2D6 genotype	—	*10*10	30 (35.71%)
*1*1	1 (1.19%)	*10*14	3 (3.57%)
*1*2	6 (7.14%)	*10*41	2 (2.38%)
*39*39	8 (9.52%)	CYP2D6 phenotype	—
*1*10	27 (32.14%)	UMs	15 (17.86%)
*1*41	3 (3.57%)	NMs	34 (40.48%)
*1*14	1 (1.19%)	IMs	35 (41.67%)
*10*34	1 (1.19%)	PMs	0 (0.00%)
*10*35	1 (1.19%)	—	—
*1*33	1 (1.19%)	—	—

### Population pharmacokinetic modeling

The number of samples collected from per patient was 1-5, and finally a total of 143 serum concentrations were collected for population pharmacokinetic modeling. Most of the blood samples were obtained after continuous administration for at least 14 days in this study, only one blood sample was collected less than 14 days because of adverse reactions. The modeling procedures were shown in Supplementary Table S1 in supplementary document. The simplest exponent model was applied as developmental model resulting from the lowest values of OFV, Akaike information criterion (AIC) and Bayesian information criterion (BIC). Comparing with the basic model, the decrease of OFV, AIC and BIC in the final model integrating with body weight and CYP2D6 genotype were 33.2, 27.2 and 16.5 units, respectively.

Both body weight and genotype were included in the final PPK model and the final model was expressed using Equations 8 to 12:

$$k_{a}\left(h^{-1}\right)=1.06$$ (8)

$$V_{d}/F\left(L\right)=219.91\times {\left(\frac{WT}{70}\right)}^{1.0}\times \exp\left(\eta_{V_{d}/F}\right)$$ (9)

$$CL/F\left(L\cdot h^{-1}\right)=3.06\times {\left(\frac{WT}{70}\right)}^{0.64}\times \exp\left(\theta_{genotype}\right)\times \exp\left(\eta_{CL/F}\right)$$ (10)

$$V_{d\left(m\right)}/F_{m}\left(L\right)=423.78\times {\left.\frac{WT}{70}\right.}^{1.0}$$ (11)

$$CL_{\left(m\right)}/F_{m}\left(L\cdot h^{-1}\right)=8.86\times {\left(\frac{WT}{70}\right)}^{0.75}\times \exp\left(\eta_{CL_{\left(m\right)}/F_{m}}\right)$$ (12)

where *WT* is the body weight, *V*_*d*_ */F* is the apparent volume of distribution of ARI, *V*_*d(m)*_ */F*_*(m)*_ is the apparent volume of distribution of DARI, *CL/F* is the apparent clearance of ARI, *CL*_*(m)*_ */F*_*(m)*_ is the apparent clearance of DARI. The clearance of ARI was decreased by 20.55% in IMs, and increased by 23.37% in UMs.

### Final model validation

**FIGURE 1:**
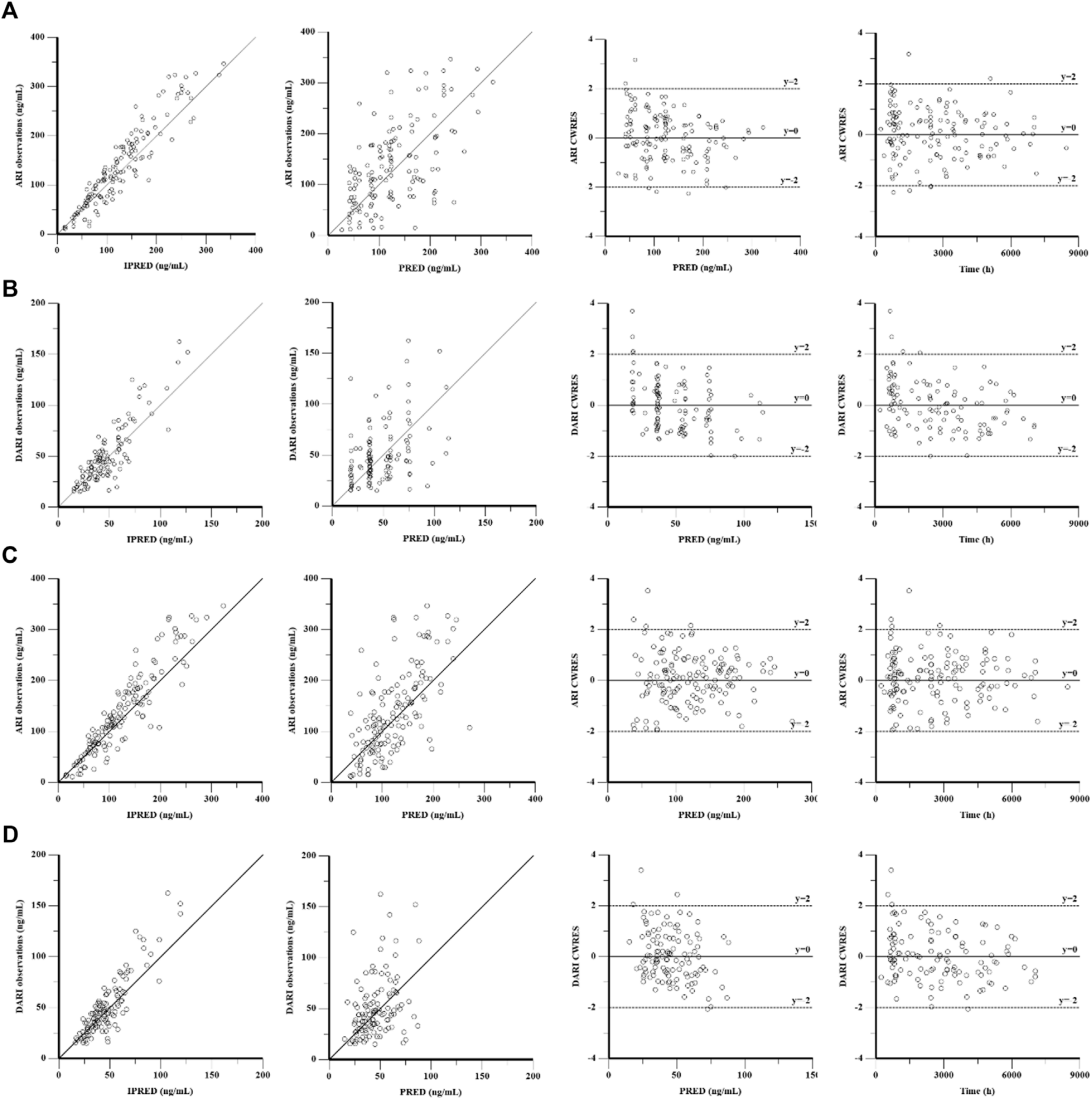
Goodness-of-fit plot of the base model and final population pharmacokinetics model for ARI **(A,C)** and DARI **(B,D)**. From left to right, the plots are observations against individual predictions (IPRED), observations against population predictions (PRED), conditional weighted residuals (CWRES) against PRED, and CWRES against time, respectively.

**FIGURE 2:**
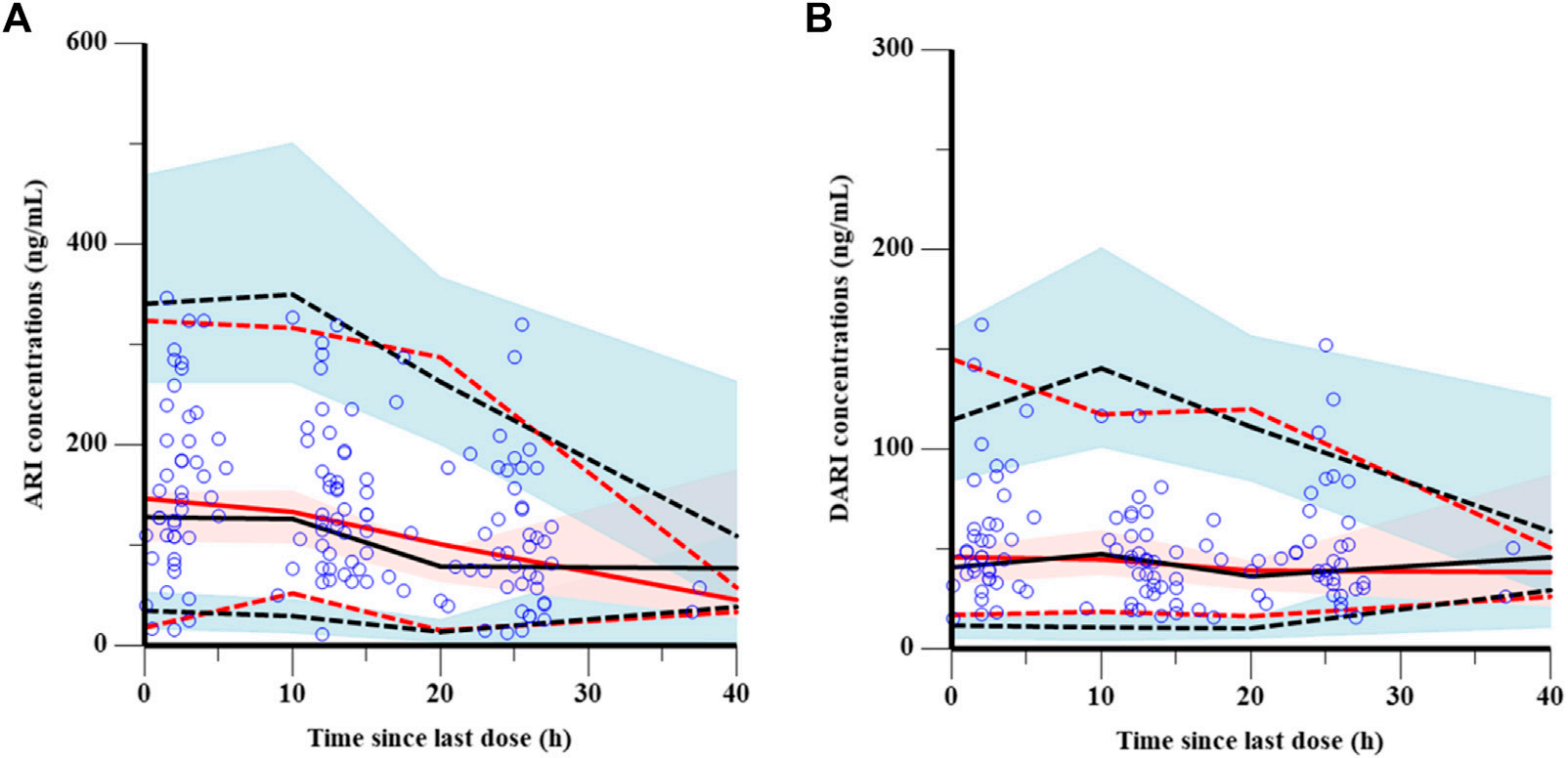
Visual prediction checks of the final model for ARI **(A)** and DARI **(B)**. The blue points represent the observed value. The dashed and solid red lines are the 5th percentile, 95th percentile and the median of the observed concentrations, respectively. The dashed and solid black lines are the 5th percentile, 95th percentile and the median of the simulated concentrations, respectively. The shaded areas represent the 90% predicted intervals of the 5th, 50th and 95th percentiles of the simulated data, respectively.

**FIGURE 3:**
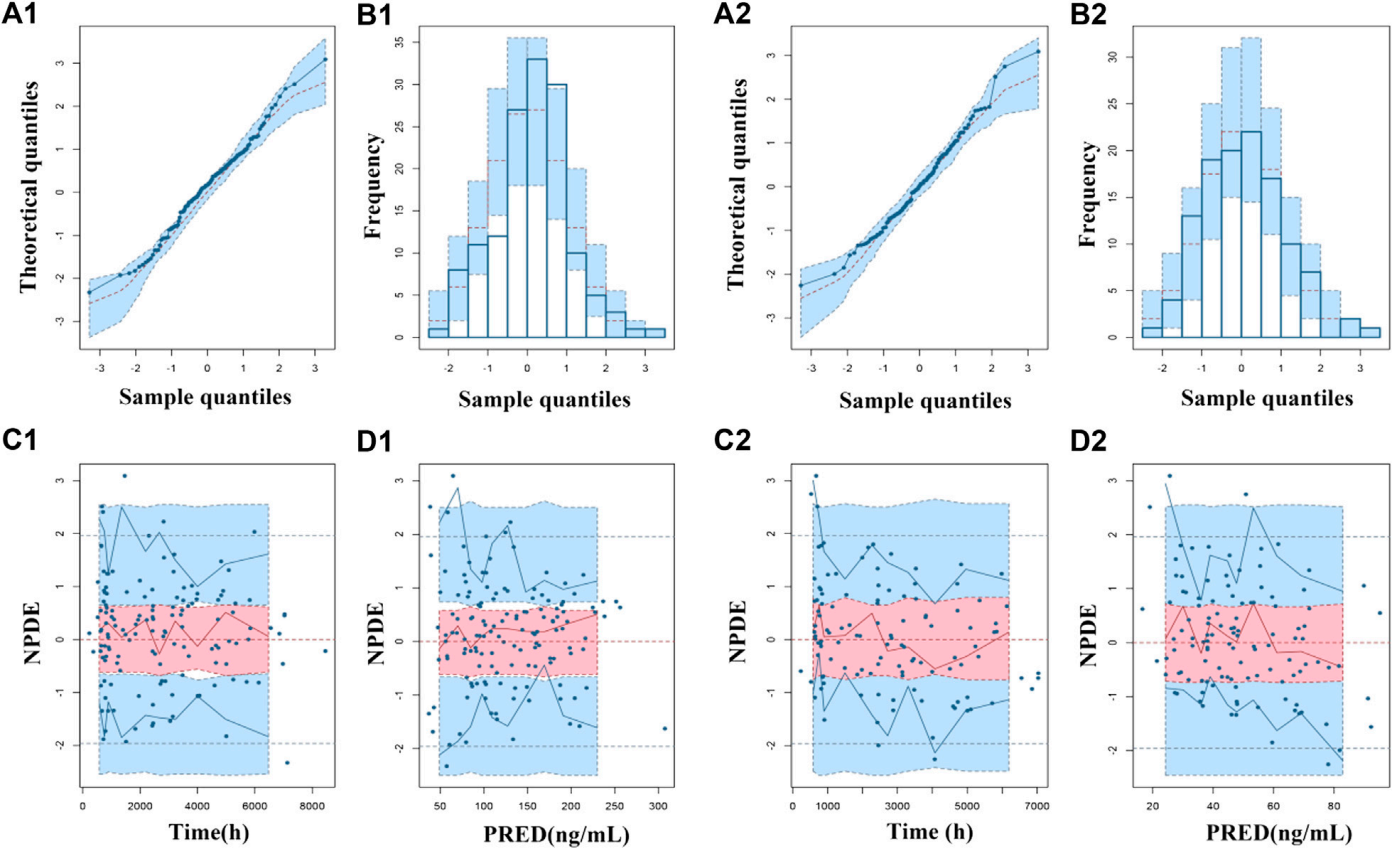
Normalized prediction distribution errors (NPDEs) of the final population pharmacokinetic model for ARI (1) and DARI (2). **(A1,A2)** Quantile-quantile plot vs. the expected standard normal distribution for ARI and DARI; **(B1,B2)** Histogram of NPDE with the density of the standard normal distribution overlaid; **(C1,C2)** Scatterplot of NPDE against time; **(D1,D2)** Scatterplot of NPDE against PRED.

The goodness-of-fit plots were presented in Figure 1. The observed serum concentrations showed closely agreement with the model prediction and conditional prediction residuals mostly located within ±2 standard deviations without significant drift, suggesting the predictive accuracy of the final model. The results of the nonparametric bootstrap analysis could be seen in Table 3. The final model estimates distributed in the 95% confidence intervals (CIs) of estimates obtained from the bootstrap procedure and were close to the median parameter estimates with small bias, which indicated good stability of the population pharmacokinetic model. Additionally, the VPCs for both ARI and DARI were shown in Figure 2. Almost all of the observed concentrations were within the 90% CIs, validating the predictive capability of the final model. As shown in Figure 3, the NPDE analysis for ARI and DARI were performed by employing t-test, Shapiro Wilks test, Fisher’s variance test, and Global test. The NPDE results were shown in Supplementary Table S2, which suggested that the NPDEs followed a normal distribution with P values larger than 0.05.

**TABLE 3:** Pharmacokinetic parameters and bootstap results of the final model.

Parameter	Final model		Bootstrap analysis	
	Estimate	RSE (%)	Bootstrap median	Bootstrap 95%CI
θ_ka_ (h^−1^)	1.06 (fixed)	—	1.06 (fixed)	—
θ_Vd/F_ (L)	219.91	23.36	226.07	158.86–370.98
θ_CL/F_ (L·h^−1^)	3.06	11.92	2.99	2.37–4.05
θ_Vd(m)/Fm_ (L)	423.78	43.01	431.19	44.47–1,097.35
θ_CL(m)/Fm_ (L·h^−1^)	8.86	5.56	8.87	7.90–9.78
θ_1_	1.00 (fixed)	—	1.00 (fixed)	—
θ_2_	0.64	21.86	0.62	0.37–0.89
θ_NM_	0.00 (fixed)	—	0.00 (fixed)	—
θ_IM_	-0.23	25.32	-0.23	-0.42–0.03
θ_UM_	0.21	35.09	0.21	0.01–0.53
θ_3_	1.00 (fixed)	—	1.00 (fixed)	—
θ_4_	0.75 (fixed)	—	0.75 (fixed)	—
Inter-individual variation				
ω^2^_Vd/F_	79.99	39.38	73.52	12.81–134.22
ω^2^_CL/F_	12.07	24.52	10.90	4.43–17.35
ω^2^_CL(m)/Fm_	15.31	30.63	14.75	5.44–24.05
Intra-individual variation				
σ_ARI_ (%)	35.45	9.34	36.10	25.72–43.17
σ_DARI_ (%)	35.37	7.17	34.66	29.38–39.44

θ_1_, exponent for WT as covariate for V_d_/F; θ_2_, exponent for WT as covariate for CL/F; θ_3_, exponent for WT as covariate for V_d(m)_/F_m_; θ_4_, exponent for WT as covariate for CL_(m)_/F_m_.

### Pharmacodynamic observations

**FIGURE 4:**
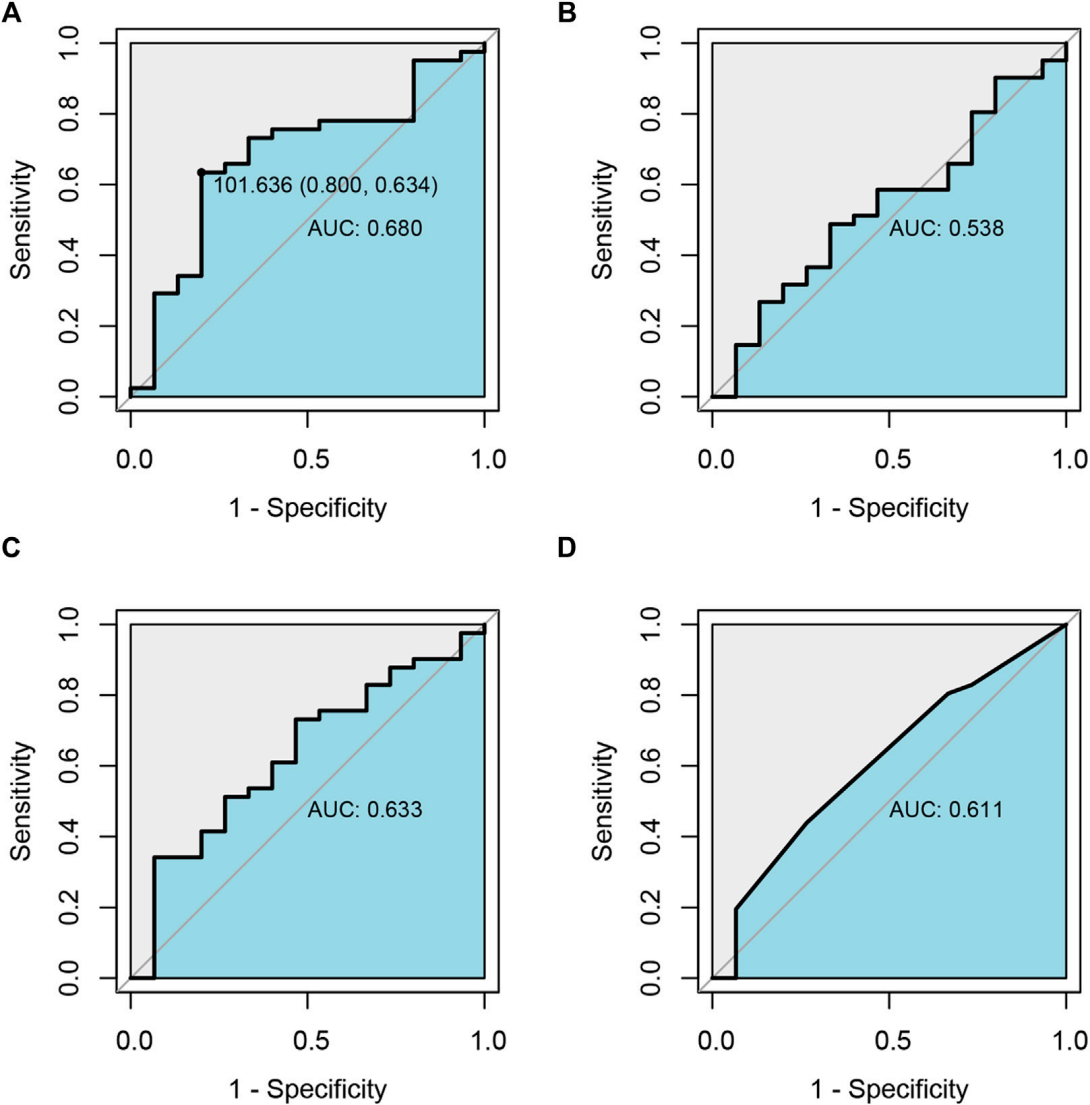
Receiver operating characteristic curve (ROC) analysis of the relationship between drug exposure of ARI**(A)**, DARI**(B)**, ARI plus DARI**(C)** and clinical efficacy, and the relationship between the dosage of ARI and clinical efficacy **(D)**.

As shown in Supplementary Table S3, totally 56 pediatric patients with TD were ultimately enrolled for the pharmacodynamic analysis of ARI. After analyzing and assessing, 73% effective rate and 26.8% adverse reaction incidence rate were acquired (41/56) after 12-week ARI monotherapy or add-on treatment. The relationships between drug exposure, the dosage of ARI and clinical efficacy were analyzed by employing ROC diagnostic curve, and the results were shown in Figure 4. The areas under the curve of ARI, DARI, ARI plus DARI and ARI dosage were 0.680, 0.538, 0.633, and 0.611, respectively. The area under the curve of ARI was the largest with the lower limit of 95%CI larger than 0.5, but the lower limits of 95%CI of DARI and ARI plus DARI were less than 0.5, suggesting the best predictor of the clinical efficacy for children with TD was the trough concentration of ARI. In addition, the cut-off point was 101.636 ng/ml, with the sensitivity and specificity of 0.800 and 0.634 respectively, which was very close to the lower limit of 100–350 ng/ml recommended by AGNP for mental disorders in adults. The intolerable adverse reactions were not observed within 350 ng/ml during the administration in this study. Therefore, 100–350 ng/ml was selected as the target index range of steady-state trough concentration of ARI.

### Simulation and dosing regimen optimization

**FIGURE 5:**
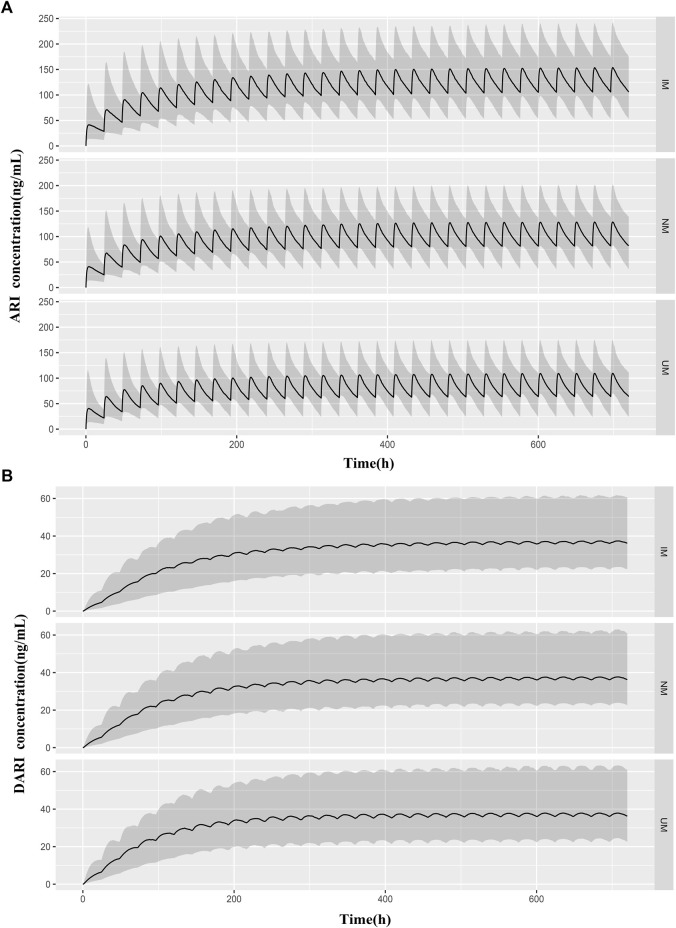
Predicted pharmacokinetic profiles of ARI **(A)** and DARI **(B)** during the first 20 days of treatment obtained from IMs, NMs, and UMs. The median body weight of 36 kg and the median dose of 0.5 mg were applied to simulated. The black line is the median of the simulated concentrations. The grey shaded area represents the prediction interval (10th-90th percentiles).

The predicted concentration-time profiles of ARI and DARI during 30 days oral administration were simulated according to the CYP2D6 phenotypes. The median body weight of 36 kg and the median dose of 0.5 mg once a day were applied to simulated. As shown in Figure 5, comparing to NMs and UMs, the trough concentrations of ARI at steady state showed significantly elevation in IMs, whereas the trough levels of DARI indicated no difference.

**FIGURE 6:**
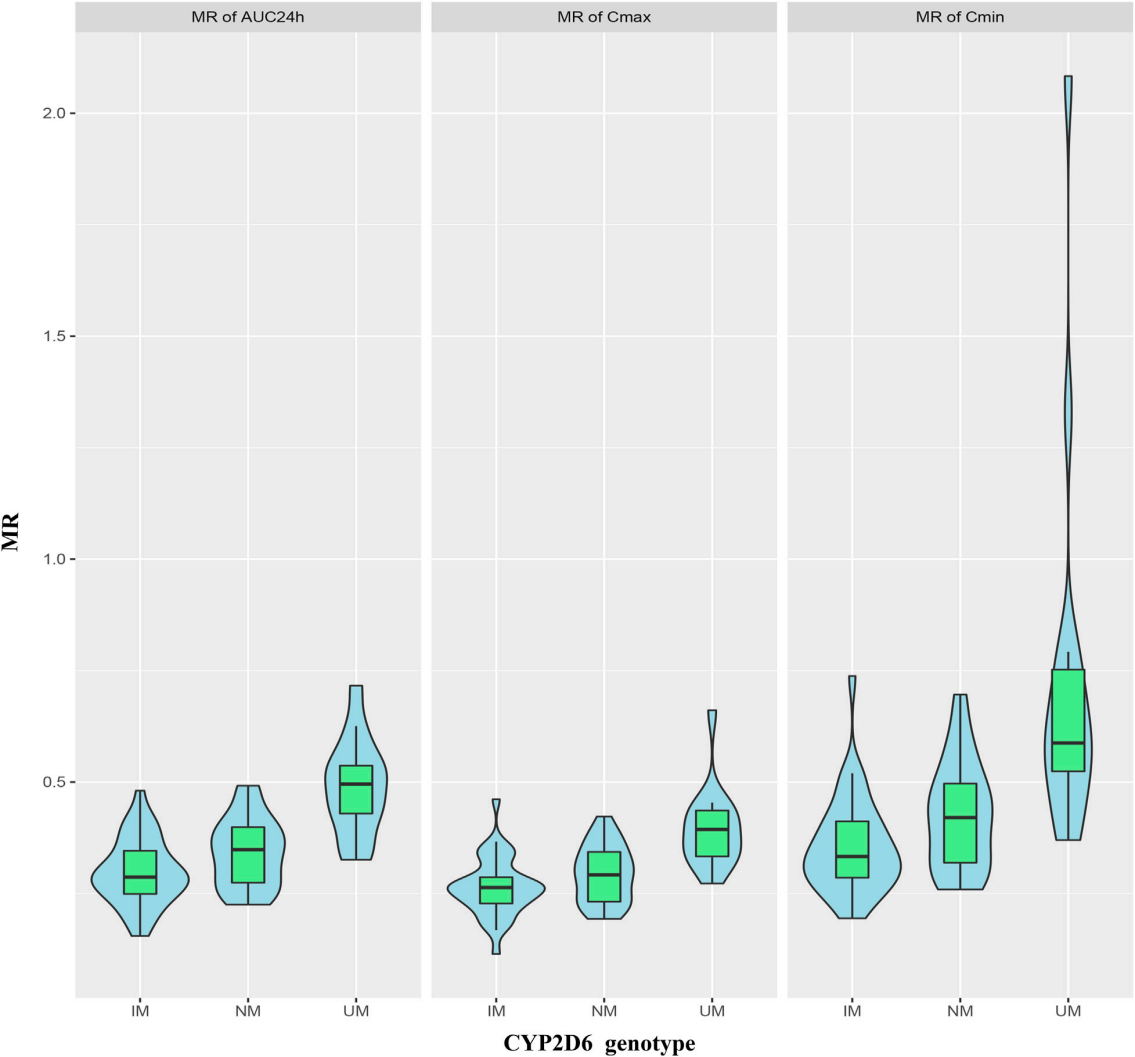
Violin pictures of DARI/ARI steady-state drug metabolic ratio (MR), including MR_AUC24h_, MR_Cmin_ and MR_Cmax_, for IMs, NMs and UMs.

The MRs of AUC_24h_, C_min_ and C_max_ for all patients were 0.35 ± 0.11, 0.45 ± 0.25 and 0.30 ± 0.08, respectively. The MRs of AUC_24h_ for UMs, NMs, IMs were 0.49 ± 0.11, 0.34 ± 0.08, and 0.30 ± 0.07; the MRs of C_min_ for UMs, NMs, IMs were 0.73 ± 0.44, 0.43 ± 0.12, and 0.36 ± 0.11; the MRs of C_max_ for UMs, NMs, IMs were 0.40 ± 0.09, 0.29 ± 0.06, and 0.26 ± 0.07, respectively. The comparison of MRs between UMs, NMs, IMs was shown in Figure 6. The violin picture showed that the MR values of AUC_24h_, C_min_ and C_max_ were UMs > NMs > IMs (*p* < 0.05). As shown in Table 4, the diagnostic cut-off points of MRs were calculated respectively. The results showed that MRs could be used to distinguish UMs or IMs from other patients.

**TABLE 4:** The diagnostic cut-off points of MRs for distinguishing UMs or IMs from other patients.

MRs	Area	95%CI	Cut-off point	Sensitivity	Specificity
MRs for UM diagnosis					
MR_AUC_	0.91 ± 0.04	0.83–0.99	≥0.42	0.80	0.90
MR_Cmin_	0.88 ± 0.05	0.79–0.97	≥0.52	0.80	0.88
MR_Cmax_	0.88 ± 0.04	0.79–0.96	≥0.32	0.93	0.71
MRs for IM diagnosis					
MR_AUC_	0.75 ± 0.05	0.64–0.85	≤0.31	0.69	0.74
MR_Cmin_	0.75 ± 0.05	0.65–0.85	≤0.40	0.71	0.69
MR_Cmax_	0.71 ± 0.06	0.60–0.83	≤0.28	0.71	0.71

**FIGURE 7:**
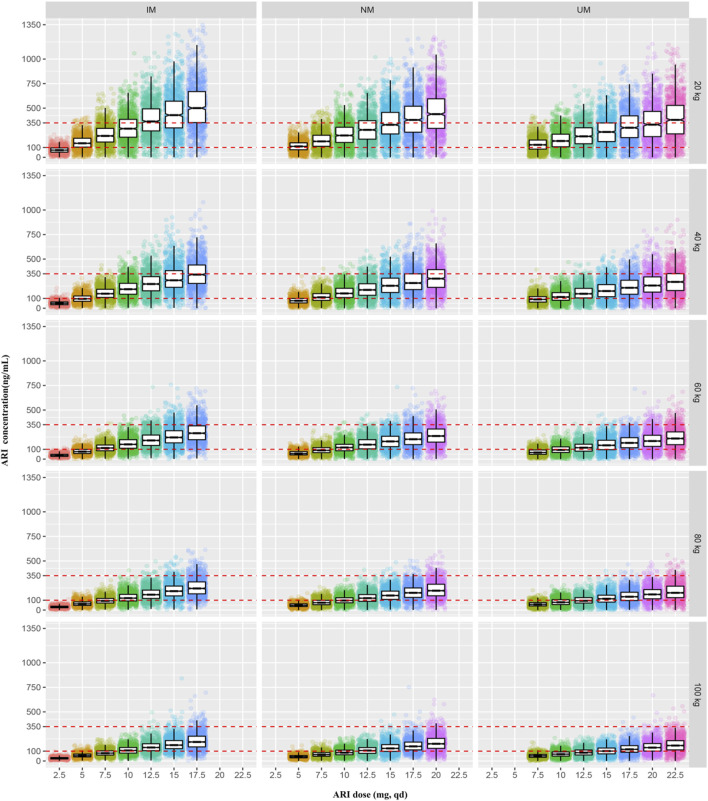
The simulated steady-state trough concentrations of ARI for patients with different body weight and CYP2D6 genotypes under the different dosage regimens.

The PTAs of the trough concentrations of ARI and ARI plus DARI under different dosage regimens for patients with different body weight and CYP2D6 genotypes were shown in Supplementary Table S4, and Figure 7 was drawn based on these results. According to the results of PTAs, we organized the optimal dosage regimens for patients with different body weight and CYP2D6 phenotypes, which was shown in Table 5. Eventually, under the optimal dosage regimens, the PTAs of C_min_ of ARI ≥100 ng/ml and C_min_ of ARI plus DARI ≥150 ng/ml were larger than 75%; the PTAs of C_min_ of ARI ≥350 ng/ml and C_min_ of ARI plus DARI ≥500 ng/ml were less than 5%; and the PTA of C_min_ of ARI ≥1,000 ng/ml was less than 1%. The results suggested that it was optimal to determine the dosing regimen of ARI for pediatric patients with TD based on body weight and CYP2D6 genotype.

**TABLE 5:** Optimal dosage regimens for patients of different CYP2D6 genotypes.

Group	Weight (kg)				
	20	40	60	80	100
NM	7.5 mg, qd	10.0 mg, qd	12.5 mg, qd	15.0 mg, qd	17.5 mg, qd
IM	5.0 mg, qd	7.5 mg, qd	10.0 mg, qd	12.5 mg, qd	12.5 mg, qd
UM	10.0 mg, qd	12.5 mg, qd	17.5 mg, qd	20.0 mg, qd	20.0 mg, qd

## Discussion

To the best of our knowledge, this research firstly described the PPK analysis of oral ARI in pediatric patients with TD. We found body weight and CYP2D6 genotype were the most important covariates that contributed to the inter-individual variability of ARI pharmacokinetics in children with TD. The study illustrated the relationship between ARI serum concentrations and clinical response, thus playing an instrumental role in proposing personalized dose regimens of ARI.

### Clinical response of ARI

In this study, the high response rate and low incidence of side effects of oral ARI reflected the good tolerance of ARI in treatment of TD. Similar findings were also found by other studies. In 2011, a prospective multicenter study on ARI against tiapride for TD suggested that the clinical response and adverse incidence rate of ARI were 60.21% and 29.6%, which paralleled with our results (Liu et al., 2011). The previous placebo-controlled clinical studies showed that oral ARI exhibited a good efficacy and safety profile for the treatment of TD (Sallee et al., 2017; Yang et al., 2019).

To date, drug exposure-efficacy relationship has not been well appreciated in pediatric TD patients. Our study confirmed a significant correlation between drug response and the parent drug (ARI) blood levels, while the correlation would be less vital when DARI or the sum of ARI and DARI were involved, which was consistent with findings from KIRSCHBAUM et al. (Kirschbaum et al., 2008). In 2011, Lin et al. (Lin et al., 2011) analysed the relationship between the serum concentrations of ARI, DARI, ARI plus DARI and clinical response in 45 patients with schizophrenia and schizoaffective disorder. Responders were found significantly higher serum concentrations of DARI and ARI plus DARI, which might be caused by the large variability of MR (DARI/ARI) in neuropsychiatric patients. The serum concentrations of ARI seem to be more significant than DARI in the dose optimization for TD patients through TDM.

### Population pharmacokinetics of ARI

Two one-compartment models were developed for both ARI and DARI to investigate the variability of pharmacokinetics of ARI. The population typical values obtained in the final PPK model were: *ka* = 1.06 h^−1^, CL/F = 3.06 L·h^−1^, Vd/F = 219.91 L. *ka* was fixed to 1.06 h^−1^ in accordance with previous researches (Kim et al., 2008; Jovanovic et al., 2020). The population typical value of CL/F in the final model was lower than the previously reported values which ranged from 3.15 L h^−1^–3.88 L·h^−1^(Kim et al., 2008; Knights and Rohatagi, 2015) but higher than that of Jeon et al.‘s report (2.69 L·h^−1^) (Jeon et al., 2016). As for the distribution volume of ARI, the Vd/F (219.91 L) obtained in this study was larger than the value of Kim et al.‘s (193 L) and Knights and Rohatagi et al.‘s (192 L) (Kim et al., 2008; Knights and Rohatagi, 2015). Racial differences, different sampling time, methods, physiological and pathological differences may contribute to the difference of the above values.

The researchers proposed that dose of ARI would not be adjusted in liver or renal impairment patients (Mallikaarjun et al., 2008). Though no liver or renal impairment occurred in this study, many other factors were taken into account. Drug combinations such as clonidine or tiapride, the most common drugs that patients used, were not included in the final PPK model in this study. Furthermore, no dose adjustments are required on the grounds of age, sex, race or smoking (Mauri et al., 2018). Moreover, gene polymorphism is deemed as a remarkable element in the field of pharmacokinetics study of ARI. The relationship between CYP2D6 genotype and ARI pharmacokinetics has been intensively probed over the past decade. Jeon et al. (Jeon et al., 2016) developed a two-compartment model for ARI in healthy Korean subjects. They proposed that CYP2D6 genotype polymorphisms, height, and weight were the covariates significantly affecting ARI pharmacokinetic parameters such as CL/F, Vc/F and Vp/F. Similarly, Knights and Rohatagi et al. (Knights and Rohatagi, 2015) found that CYP2D6 genotype, weight (<115 kg) and age were the important covariates that influenced CL/F of ARI. Regarding the young population, few population models and the pharmacokinetic analyses embraced pediatric patients. The final model established in this study showed that the clearance of ARI in pediatric TD patients could be markedly affected by body weight and CYP2D6 genotype.

### CYP2D6 and ABCB1 genetic polymorphisms in ARI

ARI metabolism is mainly mediated by CYP2D6, CYP3A4 and ABCB1 (Belmonte et al., 2018). It has been proved that CYP2D6, ABCB1 C3435T and G2677T/A but not CYP3A4 genotype were the most commonly reported genotypes associated with variability in absorption and excretion of ARI (Belmonte et al., 2018; Rafaniello et al., 2018). Furthermore, it was demonstrated that CYP3A5 polymorphism had no impact on the pharmacokinetics (Kim et al., 2008; Suzuki et al., 2014; Jeon et al., 2016). Although one study showed that subjects with CYP3A5 *3/*3 had a lower dehydro-aripiprazole/aripiprazole ratio (Belmonte et al., 2018), the influence of CYP3A5 on the pharmacokinetics of ARI and DARI is much smaller than that of CYP2D6. Therefore, CYP2D6 and ABCB1 SNPs were detected in this study. As previously reported, IMs comprise a large proportion of the Asian people, especially for CYP2D6*10 which accounts for more than 60% (Zhang et al., 2019). The IMs, NMs, UMs accounted for 41.7%, 40.5%, 17.8% respectively in this study, but no PMs were found. There was evidence that AUC_0-t_ of ARI of PMs was increased by 50% and AUC_0-t_ of DARI was decreased by 33% compared to EMs (Belmonte et al., 2018). In this study, we found that the C_min_ of ARI at steady state were notably increased in IMs in contrast to UMs and NMs, but no difference in the C_min_ of DARI. This suggested that the CYP2D6 genotype had no effect on the clearance of DARI. The critical metabolic rate-limiting step may lie in the metabolism of ARI but not in the clearance of the metabolites. At the same time, we also compared MRs of AUC_24h_, C_min_ and C_max_ between the UMs, NMs and IMs, results showed that MRs were lower in subjects with lesser active CYP2D6 alleles and this conclusion was in agreement with Belmonte et al.‘s report (Belmonte et al., 2018). MRs of AUC_24h_, C_min_ and C_max_ helped to distinguish UMs or IMs from other patients. AUC curves of MRs of AUC_24h_, C_min_ and C_max_ as predictors of IMs or UMs were calculated with high sensitivity and specificity (Table 4). These parameters conferred precious value in clinical. To some extent, we can substitute MRs for CYP2D6 genotyping, which can make up for the large costs of genetic testing in poor areas or families and further promote individual medication.

ABCB1 genes variants may alter transport activities performed by their gene products, thus to influence the serum concentrations of drugs. It was reported that the clearance of ARI in ABCB1 TT/TT subjects would be lower (Moons et al., 2011). In view of already published works, controversial conclusions have been made by many researchers. Rafaniello et al. (Rafaniello et al., 2018) investigated the impact of ABCB1 on ARI concentrations in ninety Caucasian pediatric patients, which displayed that ABCB1 2677TT/3435TT genotype had a statistically significant lower ARI serum concentration/dose ratio compared with other ABCB1 genotypes. Nevertheless, Suzuki et al. (Suzuki et al., 2014) revealed that there was no difference between ABCB1 variants (C3435T and G2677T/A) and the exposure of ARI in Japanese adult patients with schizophrenia. Five ABCB1 TT/TT subjects were detected in this study but no relationship between ABCB1 variants (C3435T and G2677T/A) and the clearance of ARI was found, possibly due to small samples of this research. Therefore, ABCB1 genetic polymorphisms were not included as significant covariates in the final model established in this study. This might be ascribed to differences in races, sample numbers or disease types of subjects in various researches. Further study is required to promote precise dose adjustment of ARI in pediatric TD patients.

### Optimal dosing regimens

So far, there is no consensus on clinical dosing regimen recommendations for ARI in tic disorders. Food and Drug Agency (FDA) or the Royal Dutch Pharmacists Association proposed that CYP2D6 PMs should be administrated 50% and 67% of maximum recommended daily dosage respectively in psychotics (Swen et al., 2011; US Food and Drug Administration, 2018). Although there are not recommendations for IMs or UMs, CYP2D6 genetic testing is still necessary in clinical dose adjustment (Bousman, 2019). The previous evidence-based researches also showed that CYP2D6 genetic testing helps to guide antipsychotic ARI medications: Kneller et al. (Kneller et al., 2021) used a physiologically based pharmacokinetic (PBPK) approach to estimate the effects of CYP2D6 phenotype-related physiological changes on the pharmacokinetics of ARI, DARI, and ARI plus DARI. They demonstrated that the daily dose should be adjusted for CYP2D6 PMs and the maximum daily dose recommended should be 10 mg. Meanwhile, the researcher proposed it was unnecessary to adjust dosage for UMs and IMs. However, the above model was developed on basis of data from healthy volunteers. Instead, IMs accounts for 45% in east Asian people, recently Jukic et al. indicated that 30% lower doses than usual should be administered to IMs and PMs (Jukic et al., 2019). This study firstly elucidated optimal and individualized dosing regimens based on different body weight and CYP2D6 genotypes in children with TD, thus ensuring therapy effectiveness and safety. For IMs, the weight of 20 kg, 40 kg, 60 kg, 80 kg, 100 kg should be given 5 mg, 7.5 mg, 10 mg, 12.5 mg, 15 mg, qd, respectively; for NMs, the weight of 20 kg, 40 kg, 60 kg, 80 kg, 100 kg should be given 7.5 mg, 10 mg, 12.5 mg, 15 mg, 17.5 mg, qd, respectively; for UMs, the weight of 20 kg, 40, 60, 80, 100 kg should be given 10, 12.5, 15, 17.5, 20 mg, qd, respectively. In China, ARI is an off-label drug for children with TD. Therefore, the individualized dosing regimens greatly help physicians to make appropriate dosing decisions.

## Limitations

The limitations of our study are as follows: 1. Due to the limited sample size, no data on PMs were obtained; 2. It was difficult to estimate pharmacokinetic parameters in absorption and distribution phase because of the limited concentration data; 3. Some of the steady-state trough concentrations of ARI and DARI were corrected according to the pharmacokinetic parameters obtained from the PPK model because the blood samples of some patients were not collected at the time point of trough concentration. Despite these limitations, our study can provide a valuable reference for personalized ARI treatment in pediatric patients with TD.

## Conclusions

This study firstly established a combined PPK model of ARI and DARI in pediatric patients with TD. The relationship between serum concentrations of ARI and clinical response was better understood and clinically important in offering a more promising therapeutic strategy for ARI. Furthermore, the influence of CYP2D6 genotype on serum concentrations of ARI, DARI, and ARI plus DARI was investigated, which offered the first proof of phenotype-weight-guided dose adjustments in pediatric TD patients. ARI has achieved beneficial clinical effects and exhibited good tolerability in the treatment of TD. Finally, we really promoted the safer, proper and more individualized dosing regimens of ARI in clinical practice.

## Data Availability

The datasets presented in this study can be found in online repositories. The names of the repository/repositories and accession number(s) can be found below: NCBI Sequence Read Archive [SRA] (https://www.ncbi.nlm.nih.gov/sra), SRR21835902.

## Glossary

**TD**,: tic disorders
**CTD**,: chronic motor or vocal tic disorders
PTD,: provisional tic disorders
TS,: Tourette syndrome
YGTSS,: Yale Global Tic Severity Scale
ARI,: Aripiprazole
**ESSTS,**: European Society for the Study of Tourette syndrome
DARI,: dehydroaripiprazole
AGNP,: Arbeitsgemeinschaft für Neuropsychopharmakologie und Pharmakopsychiatrie
TDM,: therapeutic drug monitoring
PPK,: population pharmacokinetics
DSM-5,: the fifth edition of the Diagnostic and Statistical Manual of Mental Disorders
ECG,: electrocardiogram
VPC,: visual prediction check
NPDE,: normalized prediction distribution errors
PRED,: population predictions
IPRED,: individual prediction
CWRES,: conditional weighted residuals
ROC,: receiver operating characteristic curve
PTA,: the probability of reaching the target
UMs,: ultra-rapid metabolizers
NMs,: normal metabolizers
IMs,: intermediated metabolizers
PMs,: poor metabolizers
ABCB1,: ATP-binding cassette sub-family B member 1
HPLC,: high-performance liquid chromatography
ALT,: alanine aminotransferase
AST,: aminotransferase
DBIL,: direct bilirubin
TBIL,: total bilirubin
ALB,: albumin
GLB,: globulin
γ-GT,: γ-glutamyltranspeptidase
BUN,: blood urea nitrogen
Cys-C,: serum cystatin C
Scr,: serum creatinine concentration
BSA,: serum creatinine concentrationBSA, body surface area
eGFR,: estimated glomerular filtration rate
UV,: ultraviolet
PCR,: polymerase chain reaction
FOCE ELS,: the first order conditional estimation-extended least squares
OFV,: the objective function value
MR,: metabolic ratio
**AUC**_24h_,: the area under the curve over 24 hours
**C**_max_,: peak concentration
**C**_min_,: trough concentration
AIC,: Akaike information criterion
BIC,: Bayesian information criterion
**CIs,**: confidence intervals
SNP,: single nucleotide polymorphism
P-gp,: P-glycoprotein
FDA,: Food and Drug Agency
PBPK,: physiologically based pharmacokinetic.
