# Supplementary material for: Understanding inter-individual variability in pharmacokinetics/pharmacodynamics of aripiprazole in children with tic disorders: Individualized administration based on physiological development and CYP2D6 genotypes
Source: Front Pharmacol. 2022 Dec 2;13:1048498. doi: 10.3389/fphar.2022.1048498 (PMC9755210; doi:10.3389/fphar.2022.1048498)
Supplement: Supplementary file 1 [file DataSheet1.docx]

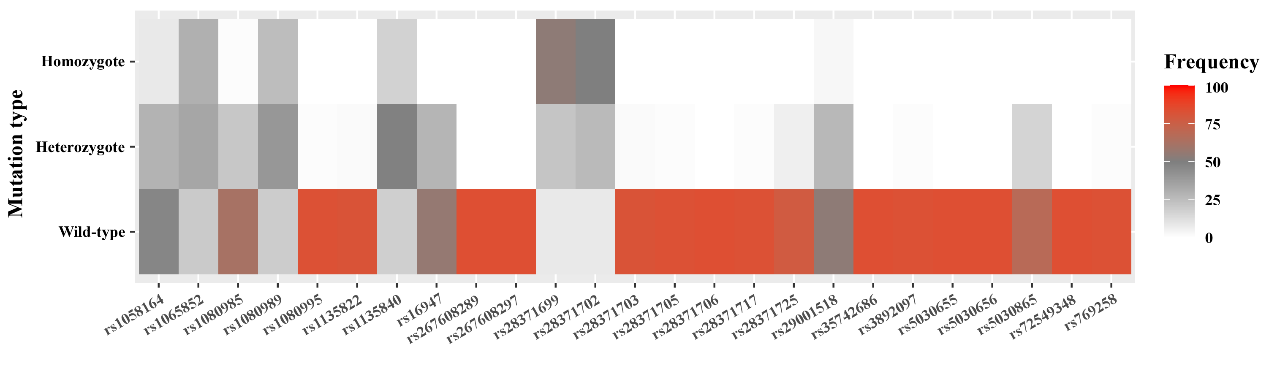


Figure S1.CYP2D6 allele mutation frequencies (Homozygote/Heterozygote/Wide-type: rs1058164 0.095/0.345/0.560;, rs1065852 0.357/0.405/0.238, rs1080989 0.298/0.476/0.238, rs1080995 0/0.012/0.988, rs1135822 0/0.024/0.976, rs1135840 0.202/0.583/0.214, rs16947 0/0.333/0.667, rs267608289 0/0/1, rs267608297 0/0/1, rs28371699 0.643/0.262/0.095, rs28371702 0.595/0.310/0.095, rs28371703 0/024/0.976, rs28371705 0/0.012/0.988, rs28371706 0/0/1, rs28371717 0/0.012/0.988 rs28371725 0/0.071/0.929, rs29001518 0.036/0.321/0.643, rs35742686 0/0/1, rs3892097 0/0.012/0.988, rs5030655 0/0/1, rs5030656 0/0/1, rs5030865 0/0.190/0.810, rs72549348 0/0/1, rs769258 0/0.012/0.988)

Table S1. The final model development process and statistical analysis

| Model description | OFV | AIC | BIC | n (parameter) |
| --- | --- | --- | --- | --- |
| **Basic model** | 2622.1 | 2640.1 | 2672.0 | 9 |
| **Basic model + Developmental model** | | | | |
| 3/4 allometric model | 2598.8 | 2616.8 | 2648.8 | 9 |
| Simple exponent model | 2598.5 | 2618.5 | 2654.1 | 10 |
| Maturation model | 2598.7 | 2620.7 | 2659.8 | 11 |
| BW-dependent model | 2598.6 | 2624.6 | 2670.8 | 13 |
| Age-dependent model | 2598.5 | 2624.5 | 2670.7 | 13 |
| **Hypothesis testing for other main**  **covariates** |  |  |  |  |
| Simple exponent model+ALT | 2594.6 | 2616.6 | 2655.7 | 11 |
| Simple exponent model+TBIL | 2598.1 | 2620.1 | 2659.2 | 11 |
| Simple exponent model+eGFR | 2597.8 | 2619.8 | 2658.9 | 11 |
| Simple exponent model+Tiapride | 2596.4 | 2618.4 | 2657.4 | 11 |
| Simple exponent model+Clonidine | 2598.2 | 2620.2 | 2659.3 | 11 |
| Simple exponent model+ALB | 2599.6 | 2621.6 | 2660.6 | 11 |
| Simple exponent model+ABCB1 | 2597.4 | 2619.4 | 2658.5 | 11 |
| **Final model** | | | | |
| Simple exponent model+CYP2D6 | 2588.9 | 2612.9 | 2655.5 | 12 |

OFV, the objective function value; AIC, akaike information criterion; BIC, bayesian information criterion; BW, body weight; ALT, alanine aminotransferase; TBIL, total bilirubin; eGFR, estimated glomerular filtration rate; ALB, albumin; ABCB1, ATP-binding cassette sub-family B member 1.

Table S2. NPDE results of the final model.

| Item | Model | |
| --- | --- | --- |
|  | ARI | DARI |
| NPDE mean (SE) | 0.133 (0.082) | 0.059 (0.094) |
| Variance (SE) | 0.946 (0.110) | 1.019 (0.130) |
| Skewness Value | 0.040 | 0.379 |
| Kurtosis Value | 0.228 | 0.085 |
| t-test p-value | 0.105 | 0.534 |
| Fisher variance test p-value | 0.671 | 0.851 |
| Shapiro-Wilks test of normality | 0.466 | 0.408 |
| Global adjusted p-value | 0.316 | 1.000 |

NPDE, normalized prediction distribution errors; SE, standard error.

Table S3. Basic information of patients of pharmacodynamics analysis in this study

|  | n | Mean ± SD | Median(range) |
| --- | --- | --- | --- |
| patients | 56 |  |  |
| Sex (male: female) | 42:14 |  |  |
| Age at baseline |  | 9.23±2.60 | 9.00 (7.92,10.58) |
| Disease type (TS: CTD) | 35:21 |  |  |
| Comorbidity |  |  |  |
| Attention deficit and hyperactivity disorder | 22 |  |  |
| Anxiety disorder | 1 |  |  |
| Depressive disorder | 1 |  |  |
| Enuresis | 2 |  |  |
| YGTSS of baseline |  | 34.04±9.46 | 32.00 (27.25,38.75) |
| YGTSS of 12 weeks |  | 13.34±8.82 | 12.00 (5.25,19.75) |

TS, Tourette syndrome; CTD, chronic motor or vocal tic disorders; YGTSS, Yale Global Tic Severity Scale.

Table S4. Simulation results of different administration schemes

| Group | Dosing regimen | Steady-state trough concentration of ARI（ng·mL^−1^） | | | |  | Steady-state trough concentration of ARI plus DARI（ng·mL^−1^） | | |
| --- | --- | --- | --- | --- | --- | --- | --- | --- | --- |
|  |  | Median | ≥100 PTA（%） | ≥350 PTA（%） | ≥1000 PTA（%） |  | Median | ≥150 PTA（%） | ≥500 PTA（%） |
| **NM** |  |  |  |  |  |  |  |  |  |
| 20 kg | 5mg, qd | 113.44 | 59.50 | 0.30 | 0.00 |  | 172.70 | 65.10 | 0.00 |
|  | 7.5 mg, qd | 170.16 | 79.30 | 4.70 | 0.00 |  | 259.05 | 88.40 | 2.50 |
|  | 10 mg, qd | 226.89 | 87.80 | 15.90 | 0.00 |  | 345.40 | 95.00 | 13.50 |
|  | 12.5 mg, bid | 283.61 | 91.00 | 31.60 | 0.10 |  | 431.75 | 97.80 | 33.60 |
|  | 15 mg, qd | 340.33 | 93.30 | 47.70 | 0.50 |  | 518.10 | 98.40 | 54.70 |
|  | 17.5 mg, qd | 397.05 | 94.50 | 59.60 | 1.30 |  | 604.45 | 99.20 | 70.50 |
|  | 20 mg, qd | 453.77 | 95.00 | 68.10 | 3.30 |  | 690.80 | 99.60 | 79.10 |
| 40 kg | 5 mg, qd | 75.65 | 24.6 | 0.00 | 0.00 |  | 111.23 | 18.10 | 0.00 |
|  | 7.5 mg, qd | 110.73 | 58.00 | 0.10 | 0.00 |  | 165.00 | 61.60 | 0.00 |
|  | 10 mg, qd | 150.41 | 79.40 | 1.00 | 0.00 |  | 225.76 | 86.00 | 0.30 |
|  | 12.5 mg, bid | 186.33 | 86.60 | 5.80 | 0.00 |  | 278.91 | 92.30 | 0.28 |
|  | 15 mg, qd | 228.40 | 91.30 | 14.30 | 0.00 |  | 338.47 | 95.80 | 10.50 |
|  | 17.5 mg, qd | 256.19 | 93.80 | 24.90 | 0.00 |  | 390.37 | 97.90 | 22.40 |
|  | 20 mg, qd | 301.03 | 94.90 | 35.60 | 0.00 |  | 443.69 | 99.00 | 36.50 |
| 60 kg | 5 mg, qd | 56.58 | 9.70 | 0.00 | 0.00 |  | 83.99 | 3.60 | 0.00 |
|  | 7.5 mg, qd | 89.34 | 38.90 | 0.00 | 0.00 |  | 130.01 | 31.10 | 0.00 |
|  | 10 mg, qd | 118.35 | 64.10 | 0.20 | 0.00 |  | 171.88 | 66.30 | 0.00 |
|  | 12.5 mg, bid | 147.43 | 77.7 | 1.20 | 0.00 |  | 213.34 | 82.70 | 0.20 |
|  | 15 mg, qd | 180.20 | 86.30 | 3.60 | 0.00 |  | 256.06 | 91.90 | 0.70 |
|  | 17.5 mg, qd | 202.44 | 90.00 | 8.50 | 0.00 |  | 296.95 | 94.90 | 4.20 |
|  | 20 mg, qd | 235.44 | 93.10 | 14.70 | 0.00 |  | 339.81 | 97.60 | 9.80 |
| 80 kg | 5 mg, qd | 48.22 | 2.10 | 0.00 | 0.00 |  | 69.49 | 0.30 | 0.00 |
|  | 7.5 mg, qd | 74.52 | 23.60 | 0.00 | 0.00 |  | 105.80 | 14.20 | 0.00 |
|  | 10 mg, qd | 96.74 | 46.30 | 0.00 | 0.00 |  | 141.44 | 41.60 | 0.00 |
|  | 12.5 mg, bid | 121.07 | 66.30 | 0.20 | 0.00 |  | 174.25 | 66.20 | 0.00 |
|  | 15 mg, qd | 148.05 | 79.00 | 1.10 | 0.00 |  | 212.99 | 82.30 | 0.30 |
|  | 17.5 mg, qd | 175.30 | 88.00 | 2.70 | 0.00 |  | 249.31 | 91.30 | 1.10 |
|  | 20 mg, qd | 197.02 | 92.30 | 7.10 | 0.00 |  | 286.88 | 96.50 | 2.40 |
| 100 kg | 5 mg, qd | 43.22 | 0.50 | 0.00 | 0.00 |  | 61.00 | 0.20 | 0.00 |
|  | 7.5 mg, qd | 64.73 | 13.5 | 0.00 | 0.00 |  | 92.05 | 5.20 | 0.00 |
|  | 10 mg, qd | 84.51 | 33.20 | 0.00 | 0.00 |  | 119.86 | 24.00 | 0.00 |
|  | 12.5 mg, bid | 106.13 | 55.50 | 0.00 | 0.00 |  | 150.57 | 50.60 | 0.00 |
|  | 15 mg, qd | 128.08 | 72.00 | 0.30 | 0.00 |  | 183.46 | 74.30 | 0.10 |
|  | 17.5 mg, qd | 148.59 | 83.20 | 1.30 | 0.00 |  | 213.53 | 85.90 | 0.30 |
|  | 20 mg, qd | 174.82 | 87.50 | 3.40 | 0.00 |  | 245.69 | 91.20 | 1.10 |
| **IM** |  |  |  |  |  |  |  |  |  |
| 20 kg | 2.5 mg, qd | 72.95 | 22.10 | 0.00 | 0.00 |  | 104.01 | 11.80 | 0.00 |
|  | 5 mg, qd | 142.42 | 76.70 | 1.60 | 0.00 |  | 203.26 | 78.20 | 0.10 |
|  | 7.5 mg, qd | 217.49 | 90.50 | 11.80 | 0.00 |  | 309.58 | 94.30 | 5.50 |
|  | 10 mg, qd | 291.25 | 93.00 | 33.1 | 0.10 |  | 410.05 | 97.00 | 27.90 |
|  | 12.5 mg, bid | 364.68 | 96.40 | 54.30 | 0.40 |  | 521.96 | 98.80 | 54.60 |
| 40 kg | 15 mg, qd | 429.05 | 96.50 | 66.40 | 1.30 |  | 610.58 | 99.00 | 69.90 |
|  | 17.5 mg, qd | 500.49 | 97.30 | 75.80 | 3.50 |  | 715.43 | 99.70 | 83.40 |
|  | 2.5 mg, qd | 49.38 | 2.70 | 0.00 | 0.00 |  | 67.05 | 0.40 | 0.00 |
|  | 5 mg, qd | 95.49 | 45.20 | 0.00 | 0.00 |  | 131.20 | 32.50 | 0.00 |
|  | 7.5 mg, qd | 147.51 | 80.00 | 0.40 | 0.00 |  | 202.08 | 80.20 | 0.00 |
|  | 10 mg, qd | 193.12 | 89.90 | 6.60 | 0.00 |  | 264.29 | 92.20 | 1.60 |
|  | 12.5 mg, bid | 246.16 | 93.20 | 17.7 | 0.00 |  | 334.62 | 96.30 | 10.30 |
|  | 15 mg, qd | 284.15 | 96.40 | 33.20 | 0.00 |  | 399.52 | 98.40 | 23.40 |
| 60 kg | 17.5 mg, qd | 342.10 | 97.10 | 47.10 | 0.10 |  | 460.95 | 99.10 | 41.00 |
|  | 2.5 mg, qd | 36.08 | 0.40 | 0.00 | 0.00 |  | 49.69 | 0.00 | 0.00 |
|  | 5 mg, qd | 76.06 | 23.60 | 0.00 | 0.00 |  | 103.14 | 10.00 | 0.00 |
|  | 7.5 mg, qd | 113.11 | 62.20 | 0.20 | 0.00 |  | 152.79 | 52.30 | 0.00 |
|  | 10 mg, qd | 150.23 | 79.70 | 1.10 | 0.00 |  | 201.54 | 79.10 | 0.00 |
|  | 12.5 mg, bid | 190.21 | 90.20 | 5.60 | 0.00 |  | 254.06 | 92.50 | 0.70 |
|  | 15 mg, qd | 220.83 | 93.00 | 11.70 | 0.00 |  | 300.13 | 95.70 | 5.20 |
|  | 17.5 mg, qd | 263.74 | 96.60 | 21.40 | 0.00 |  | 353.56 | 98.30 | 11.70 |
| 80 kg | 2.5 mg, qd | 30.73 | 0.10 | 0.00 | 0.00 |  | 41.26 | 0.00 | 0.00 |
|  | 5 mg, qd | 63.22 | 12.40 | 0.00 | 0.00 |  | 84.05 | 3.20 | 0.00 |
|  | 7.5 mg, qd | 93.40 | 42.10 | 0.00 | 0.00 |  | 125.90 | 25.90 | 0.00 |
|  | 10 mg, qd | 121.46 | 68.90 | 0.10 | 0.00 |  | 164.94 | 59.70 | 0.00 |
|  | 12.5 mg, bid | 156.58 | 84.30 | 1.20 | 0.00 |  | 209.28 | 83.40 | 0.30 |
|  | 15 mg, qd | 192.33 | 92.10 | 4.30 | 0.00 |  | 255.28 | 92.60 | 1.20 |
|  | 17.5 mg, qd | 219.90 | 96.40 | 10.70 | 0.00 |  | 296.67 | 97.00 | 3.60 |
| 100 kg | 2.5 mg, qd | 27.34 | 0.00 | 0.00 | 0.00 |  | 36.23 | 0.00 | 0.00 |
|  | 5 mg, qd | 54.52 | 4.80 | 0.00 | 0.00 |  | 72.33 | 0.70 | 0.00 |
|  | 7.5 mg, qd | 80.03 | 27.70 | 0.00 | 0.00 |  | 106.22 | 12.60 | 0.00 |
|  | 10 mg, qd | 106.82 | 56.70 | 0.00 | 0.00 |  | 141.78 | 42.30 | 0.00 |
|  | 12.5 mg, bid | 137.20 | 77.50 | 0.50 | 0.00 |  | 180.91 | 72.30 | 0.10 |
|  | 15 mg, qd | 161.51 | 87.30 | 2.10 | 0.00 |  | 215.14 | 87.20 | 0.50 |
|  | 17.5 mg, qd | 193.70 | 92.40 | 4.90 | 0.00 |  | 256.19 | 92.50 | 1.50 |
| **UM** |  |  |  |  |  |  |  |  |  |
| 20 kg | 7.5 mg, qd | 126.72 | 66.30 | 1.00 | 0.00 |  | 220.17 | 81.30 | 0.30 |
|  | 10 mg, qd | 165.76 | 77.70 | 5.90 | 0.00 |  | 290.44 | 91.70 | 4.90 |
|  | 12.5 mg, bid | 212.77 | 84.80 | 14.40 | 0.00 |  | 366.19 | 96.10 | 17.50 |
|  | 15 mg, qd | 257.53 | 87.20 | 24.80 | 0.00 |  | 438.08 | 97.00 | 33.90 |
|  | 17.5 mg, qd | 300.60 | 91.80 | 37.50 | 0.00 |  | 514.55 | 98.40 | 53.60 |
|  | 20 mg, qd | 332.11 | 90.2 | 46.50 | 0.60 |  | 574.27 | 99.10 | 63.80 |
|  | 22.5 mg, qd | 380.69 | 92.20 | 55.20 | 1.30 |  | 654.14 | 99.30 | 75.70 |
| 40 kg | 7.5 mg, qd | 89.37 | 40.10 | 0.10 | 0.00 |  | 143.85 | 45.20 | 0.00 |
|  | 10 mg, qd | 113.81 | 60.50 | 0.20 | 0.00 |  | 188.33 | 73.10 | 0.10 |
|  | 12.5 mg, bid | 146.71 | 76.70 | 1.10 | 0.00 |  | 240.87 | 88.30 | 0.60 |
|  | 15 mg, qd | 174.58 | 82.60 | 4.80 | 0.00 |  | 285.49 | 91.80 | 3.70 |
|  | 17.5 mg, qd | 207.78 | 86.90 | 11.20 | 0.00 |  | 337.84 | 95.30 | 10.50 |
|  | 20 mg, qd | 230.73 | 90.20 | 18.80 | 0.00 |  | 381.04 | 97.60 | 19.80 |
|  | 22.5 mg, qd | 267.64 | 91.40 | 25.60 | 0.00 |  | 429.20 | 98.60 | 30.50 |
| 60 kg | 7.5 mg, qd | 67.29 | 19.60 | 0.00 | 0.00 |  | 109.54 | 17.60 | 0.00 |
|  | 10 mg, qd | 93.72 | 44.60 | 0.00 | 0.00 |  | 147.51 | 48.5 | 0.00 |
|  | 12.5 mg, bid | 116.35 | 62.10 | 0.20 | 0.00 |  | 182.71 | 72.2 | 0.00 |
|  | 15 mg, qd | 140.58 | 73.10 | 1.00 | 0.00 |  | 221.10 | 83.70 | 0.30 |
|  | 17.5 mg, qd | 165.20 | 81.40 | 2.20 | 0.00 |  | 258.59 | 91.90 | 0.70 |
|  | 20 mg, qd | 184.12 | 84.80 | 6.10 | 0.00 |  | 290.53 | 93.50 | 4.00 |
|  | 22.5 mg, qd | 209.79 | 89.20 | 9.50 | 0.00 |  | 327.92 | 96.50 | 7.40 |
| 80 kg | 7.5 mg, qd | 57.74 | 8.20 | 0.00 | 0.00 |  | 89.03 | 4.00 | 0.00 |
|  | 10 mg, qd | 79.03 | 30.20 | 0.00 | 0.00 |  | 121.43 | 26.00 | 0.00 |
|  | 12.5 mg, bid | 94.06 | 45.90 | 0.00 | 0.00 |  | 150.98 | 51.10 | 0.00 |
|  | 15 mg, qd | 114.78 | 61.10 | 0.20 | 0.00 |  | 179.09 | 69.50 | 0.00 |
|  | 17.5 mg, qd | 135.53 | 73.20 | 0.80 | 0.00 |  | 213.53 | 82.10 | 0.30 |
|  | 20 mg, qd | 159.26 | 82.30 | 1.60 | 0.00 |  | 246.10 | 89.70 | 0.80 |
|  | 22.5 mg, qd | 175.41 | 86.50 | 4.10 | 0.00 |  | 277.71 | 94.90 | 1.90 |
| 100 kg | 7.5 mg, qd | 51.65 | 3.20 | 0.00 | 0.00 |  | 79.37 | 0.90 | 0.00 |
|  | 10 mg, qd | 69.43 | 18.60 | 0.00 | 0.00 |  | 105.08 | 12.30 | 0.00 |
|  | 12.5 mg, bid | 84.52 | 33.00 | 0.00 | 0.00 |  | 129.06 | 34.30 | 0.00 |
|  | 15 mg, qd | 100.72 | 50.90 | 0.00 | 0.00 |  | 156.56 | 54.70 | 0.00 |
|  | 17.5 mg, qd | 118.55 | 65.80 | 0.20 | 0.00 |  | 182.53 | 73.60 | 0.00 |
|  | 20 mg, qd | 136.00 | 76.10 | 0.90 | 0.00 |  | 211.20 | 85.30 | 0.20 |
|  | 22.5 mg, qd | 157.21 | 81.30 | 2.00 | 0.00 |  | 239.76 | 89.80 | 0.50 |

PTA, the probability of reaching the target; UM, ultra-rapid metabolizer; NM, normal metabolizer; IM, intermediated metabolizer; PM, poor metabolizer.
